# Characterization and Evaluation of *Metarhizium* spp. (Metsch.) Sorokin Isolates for Their Temperature Tolerance

**DOI:** 10.3390/jof8010068

**Published:** 2022-01-10

**Authors:** Viswakethu Velavan, Rajendran Dhanapal, Govindaraju Ramkumar, Sengodan Karthi, Sengottayan Senthil-Nathan, Osmund A. Ndomba, Eliningaya J. Kweka

**Affiliations:** 1 ICAR-National Bureau of Agricultural Insect Resources, Bengaluru 560024, India; 2 Ashoka Trust for Research in Ecology and the Environment (ATREE), Bengaluru 560064, India; 3 Adhiparasakthi Horticultural College, Tamil Nadu Agricultural University, Ranipet 632506, India; 4 Department of Entomology, Banaras Hindu University, Varanasi 221005, India; 5 Sri Paramakalyani Centre for Excellence in Environmental Sciences, Division of Bio pesticides and Environmental Toxicology, Manonmaniam Sundaranar University, Tirunelveli 627012, India; ayvidram@gmail.com (G.R.); karthientomology@gmail.com (S.K.); 6 Division of Livestock and Human Diseases Vector Control, Tropical Pesticides Research Institute, P.O. Box 3024, Arusha 23xxx, Tanzania; ndomba@tpri.go.tz (O.A.N.); pat.kweka@gmail.com (E.J.K.); 7 Department of Medical Parasitology and Entomology, School of Medicine, Catholic University of Health and Allied Sciences, P.O. Box 1464, Mwanza 33xxx, Tanzania

**Keywords:** *Metarhizium anisopliae*, *Metarhizium robertsii*, *Metarhizium majus*, *Metarhizium quizhouense*, chitinase, protease, temperature tolerance, *Hyblaea puera*

## Abstract

A field survey was done in teak (*Tectona grandis* F.) forests in South India to explore the entomopathogenic effect of *Metarhizium anisopliae* (Ascomycota: Sordariomycetes) against teak defoliator, *Hyblaea puera* (Lepidoptera: Hyblaeidae). About 300 soils and infected insect samples were collected during the survey and thirty-six fungal isolates were isolated from soil and insect samples and characterized. The fungi were cultured on PDAY with dodine and antibiotics. Generally, the EPF culture was incubated at 27 °C in darkness for 15 days. Virulence of the Entomopathogenic Fungi (EPF) ability to germinate under cold and heat temperatures was assessed in a culture impregnated with conidia. In the experiment, it was found that for the first time *Metarhizium quizhouense*, *Metarhizium robertsii*, and *Metarhizium majus* species caused significantly higher mortality to hosts. These isolates of *M. anisopliae, M. robertsii, M. majus*, and *M. quizhouense* were all considered to be effective virulent and environmentally adaptive. The *Metarhizium* isolates were recommended as effective bio-control agents through the field investigation of teak defoliator *Hyblaea puera* from South India forest. This study paves the way to utilize the indigenous isolates of EPF for the control of teak defoliator and to combat the pests thatare resistant to insecticide.

## 1. Introduction

Entomopathogenic fungi (EPF) are widely used as bio-agents against various insect pests in agriculture, forest, other crop pests, and aquatic invertebrates. For about 100 years, myco-insecticides have been established [1] as one of the most promising pest biocontrol options in important insects or other arthropods [2,3]. However, it increases the low growth rate of opportunistic fungi [4,5,6].

The use of EPF as a pest management tool is affected by many biotic and abiotic factors. During conidia germination, they are affected by enhanced activity of the secondary metabolism [7,8,9,10,11]. Thermotolerance is an important factor that influences the survival, growth, development, and pathogenicity of EPF [12]. The *Metarhizium* sp., culture showed delayed conidial germination and mycelia growth when it was exposed to lower light [13,14]. Conversely, the EPF tolerated heat temperature during mycelia growth, which induces the high-protein metabolism [15,16].

The EPF isolates have been an asset in pest control because of their contact mode of action, the production of toxins in the insect body. When the conidia makes contact with the insect integument, they can directly penetrate insects’ pest bodies through the cuticle degradation enzyme [17,18,19] by the development of endo-chitinase, chitin deacetylase, chitosanase, alkaline protease, and lipase [20,21], which facilitate infection. In previous studies, it was described that *M. anisopliae* isolates were one of the major virulent entomopathogenic microorganisms that induced large amounts of extracellular enzymes (chitinase, lipase, and protease) for penetrating host cuticles [22,23].

The effect of the biocontrol agents was tested against various insect orders, with the case study being an important forest pest called teak defoliator, *Hyblaea puera* Cramer (Hyblaeidae). The defoliator is considered as a very serious pest in both forest plantations and natural forests [24]. It is a host specific moth that is native to Southeast Asia [25]. First described by Pieter Cramer, caterpillars of the species have been recently reported to feed on teak and other high value trees [26,27] in India. In vitro screening of an entomopathogenic fungus called *Metarhizium* spp. was done against *Hyblaea puera*. Out of the nine isolates of the EPF (*Metarhizium* sp.), four species of *M. anisopliae*, *M. robertsii*, *M. majus*, and *M. quizhouense* were analyzed by testing conidia produced under light and dark conditions to detect any differences in conidia thermo-tolerances. The best performing EPF isolate was described as being the potential in pest control, which plays an important role in secondary metabolism, caused by several insect groups. In the current research, an investigation of EPF isolates found in forest soils and infected insects and their pathogenicity against teak defoliator was done. This is one of the pioneer experiments investigating a novel way to use *M. robertsii*, *M. majus*, and *M. quizhouense* for the biological control of teak defoliator *Hyblaea puera*.

## 2. Materials and Methods

### 2.1. Fungal Isolates and Culture Conditions

**Figure 1:**
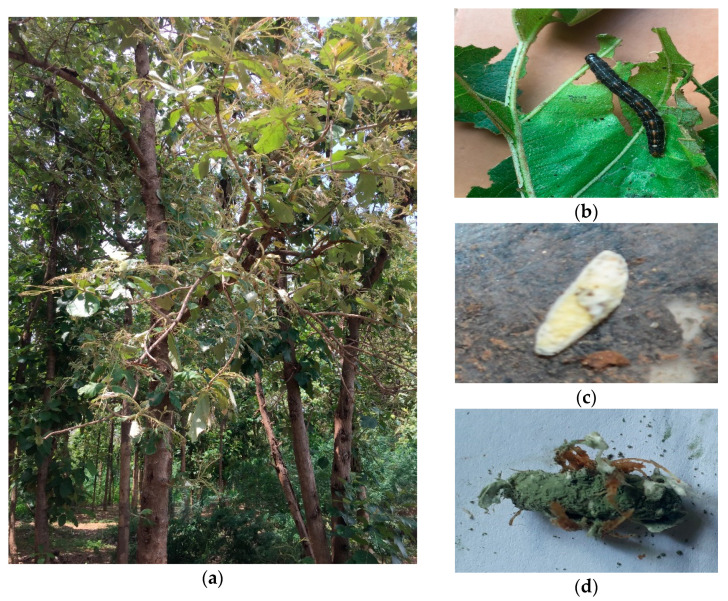
(**a**) Teak forest with *H. puera* infestation in field, (**b**) *H. puera* feeding on leaves, (**c**) Dead larvae showing the symptoms of *M. quizhouense* infection, and (**d**) Insect mummified covered *M. robertsii* infected cadaver with spores.

During the survey, 300 soil samples were collected from 20 different climatic locations of South India, and 16 infected cadavers were collected [28] (Figure 1). The fungus was isolated from the soil using the *Galleria* bait method. The fungal infected was subsequently purified on Veen’s medium containing [29] Dodine, subjected to selective media PDAY supplemented with antibiotics with the following composition (1% yeast extract (0.6 g), Chloramphenicol (100 μg/mL), Streptomycin (50 μg/mL), crystal violet (2 mg) in Potato Dextrose Agar with Yeast extract (PDAY), and then incubated at 27 ± 2 °C for up to 5 to 14 days with RH 80% to evaluate the fungal viability. EPF isolates were continuously sub-cultured on Sabouraud Dextrose Agar with Yeast extract (SDAY) slants at 28 °C and maintained at 4 °C. Further, microscopic permanent slide cultures were prepared for the identification of the *Metarhizium* sp. [30].

### 2.2. Insect Culture

The insect cultures of teak defoliator (*Hyblaea puera*) were maintained at ICFRE-Institute of Wood Science and Technology, Bengaluru, Karnataka, India. The culture was maintained on the natural host plant (teak) for two generations. For establishing the insect culture, larvae were grown on fresh tender teak leaf. The leaves were collected and stored in the plastic box that contained moist filter paper disc. The leaves were changed every 10 days. After establishment of the culture under laboratory conditions, the larvae were maintained on artificial media. The larvae were reared on ingredients of artificial diet (Kabuligram flour, Multivitamin and mineral mixture, Vitamin E, Streptomycin sulphate, Tetracycline hydrochloride, Sorbic acid, Ascorbic acid, and Formaldehyde) under laboratory conditions [31]. Individual larvae were then reared separately in sterilized plastic tubes (7 cm × 2.5 cm) [25]. The cups were covered with aluminum foil to prevent the larvae from escaping. The culture was maintained at the temperature at 28 ± 2 °C and 75 ± 5% RH.

### 2.3. Bioassay of Metarhizium spp. against Hyblaea puera

Out of the 36 isolates that were screened, nine EPF isolates were evaluated through the YPD medium (0.2% yeast extract, 1% peptone, 2% dextrose with 1% casein/10 g chitin purified powder (25 ± 2 °C, 75 ± 5% RH) incubated at 15 days post inoculation and some modification in Pontecorvo [32]. Based on the preliminary pathogenicity studies, the virulent isolates of nine *Metarhizium* sp. were used for the bioassay against 3rd instar larvae of *H. puera*, using for the concentration of 1 × 10^3^ to 1 × 10^6^ conidial/mL to determine their pathogenicity. Each experiment was replicated three times. The conidial suspension was prepared using the 15 days old culture by scrapping the mycelial growth in sterile water and then filtering in double layer muslin cloth. The stock conidial suspension was prepared by counting the conidia using the improved Neubauer Heamocytometer. Uniform dispersion of conidial suspension was achieved by adding the wetting agents (0.2% Tween-80) to the stock solution. The teak leaf was dipped in the conidial suspension and provided for feeding to the 3rd instars larvae. In each replication, 15 larvae were used. The mortality counts were taken daily for 10 days. The dead larvae were placed on moist filter paper in Petri plates.

### 2.4. Production of Cuticle-Degradation Enzymes

To obtain the insect cuticle, infected 3rd insect larvae of the *H. puera* cuticles were used. The internal materials of the dead insect larvae were removed and dried to a constant weight in an oven at 80 °C. Then, the exoskeleton was macerated. The resulting powder was sieved and stored at −20 °C. At the time of experiment, the powder was suspended in an aqueous solution of potassium tetraborate (1%). The extract was subjected to a flowing steam for 20 min [33], dried in a sterile-air-flow cabinet to avoid contamination, and inoculated with 50 µL distilled water, which contained 5000 conidia. Following incubation (60 h) at 27 ± 5 °C, the cuticles were checked microscopically to ensure there was no bacterial contamination and enzymes were extracted as described [33]. The cuticle extract was placed on performed enzyme specific medium and incubated at 27 °C for 15 days after growth of the EPF species.

### 2.5. Estimation of Extracellular Enzyme Activity on H. puera

Among the nine isolates selected, *Metarhizium* spp. was grown in a selective medium for 12 days as described above. Protease activity was measured using casein as a substrate 0.6% (*w*/*v*). Casein solution (prepared by mixing 6 mg/mL casein with the reaction mixture) contained 100 µL of Tris-HCl buffer (20 mM, pH 8) and was inoculated with 1 mL of the enzyme at 30 °C for 10 min [22,23]. A measure of 0.5 mL of the supernatant was added (1.25 mL of 0.4 M Na_2_CO_3_ and 0.25 mL of 2N Folin). It was then added to 0.4 M trichloroacetic acid (TCA) solution and the absorbance was measured at 650 nm in a spectrophotometer, which was incubated at 30 °C for 30 min. The amount of amino acids released was calculated from a standard curve plotted against a range of known concentrations of tyrosine. Afterwards, the product was centrifuged for 5 min at 10,000 rpm. One unit of enzyme was defined as the amount of enzyme that released 1 μg of tyrosine mL^−1^ of crude enzyme.

Chitinase enzyme activity of the culture supernatant was estimated using the substrate acid-swollen chitin [20,34]. To prepare the acid-swollen chitin, 10 g of chitin (purified powder from crab shells (HiMedia, Mumbai, Maharashtra) was suspended in 100 mL of HCl (35%, *w*/*v*) and the mixture was stirred at 5 min intervals for 1 h at room temperature in a fume hood. One unit of chitins was defined as the amount of enzyme that released N-acetyl D-glucosamine, or 100 µL dinitrosalicylic acid was added with incubation for 10 min inboiling water. Absorbance of the reaction mixture at 582 nm (A582) was measured after cooling the room temperature [35]. The procedure is described above, and the bioassay was conducted according to [32].

### 2.6. Temperature Optimization of Fungal Isolates Growth

#### 2.6.1. Effect of Heat Temperature on Relative Germination (RG)

The relative germination of EPF at high temperature (45 °C & 48 °C) was checked on the 9 virulent isolates of *Metarhizium* spp., Out of 36 isolates, nine isolates has been found to be virulent isolates. The EPF was cultured on PDAY medium and kept in the dark at 27 ± 1 °C for 15 days. Mycelium of the fungus was harvested with a microbiological loop in sterile water and immediately suspended in Tween 80 solution. The suspension was filtered out through double layer muslin cloth to get conidia. The conidial concentration was prepared by counting the conidia under the Neubauer hemocytometer and the concentration of 10^6^ conidia/mL was made. The 2 mL filtered suspension was transferred to a Borosil glass screw cap tube and kept in water bath at 45 °C and 48 °C for 5 ± 1 min. After 2, 4, 6, 8, 10, 12, 24, 36, 48, and 60 h of exposure with 45 °C and 48 °C, a 20 µL aliquot was placed on 4 mL of medium (PDAY) and one drop of lactophenol methyl blue was added, after which a coverslip was placed on it [36]. The germination was observed at 400× magnification. For the control, the conidial suspension was not exposed at 45 °C and 48 °C temperatures. All of the culture was incubated at 27 ± 2 °C. The observation was taken on the 300 conidia and the germination percentage was calculated by comparing the germination of the heated conidia with the control [37]. Only germinated isolates were taken for assessment.

#### 2.6.2. Effect of Cold Temperature on Relative Germination (RG)

To know the effect of cold temperature (5 °C, 10 °C, and 20 °C) on relative germination, the 2 mL of filtered suspension was transferred to a (Borosil glass) screw cap tube and exposed to cold temperature at 5 °C, 10 °C, and 20 °C. After 5, 10, and 15 days of exposure, 20 µL of conidial suspension of each isolate was poured on 4 mL of medium by following the procedure that was used for the effect of heat temperature. For the control, the conidia were grown in ambient temperature (28 ± 2 °C) without exposure to cold temperature [38].

### 2.7. Analysis of Results

Significant mortality of the larvae (percent) was recorded from the bioassay and corrected according to Abbott’s formula. Two-way ANOVA was used to compare the effects of the experimental and control treatments on the larval mortality. Prior to statistical analysis, data expressed as proportions were subjected to a regular arc transformation (one way analysis *p* < 0.001). The data were then subjected to analysis of variance (ANOVA), and the means were compared by Tukey test at the (*p* < 0.001) probability level using (Graph was generated using Origin Lab Professional Version 2021b software; URL link: www.OriginLab.com/2021b, accessed on 29 September 2021).

## 3. Results

### 3.1. Isolation and Identification of Fungi

A total 36 isolates were obtained from forest soils and infected cadavers, which were collected from various locations [39]. There were four species of entomopathogenic fungi, viz., *M. robertsii*, *M. quizhouense*, *M. majus,* and *M. anisopliae,* from the soil and infected cadaver sample (Table 1). At the same time, other entomopathogenic fungi were grown in culture medium but failed to infect the host insects and were, therefore, not taken for further study. Moreover, during the isolation on PDAY culture medium, opportunistic fungi, viz., *Aspergillus*, *Penicillium, Colletotrichum*, and *Scopulariopsis,* were dominant and these fungi were not considered for further study in this identification. All these isolates were molecularly characterized using ribosomal internal transcribed spacer region (ITS) and DNA-directed RNA polymerase II subunit (RPB1) [39], and the Elongation factor 1-alpha (EP1α) genes sequence was analyzed as well, although it could not yet be submitted (NCBI).

### 3.2. Bioassay Tested Metarhiziumsp. Isolates against H. puera

**Figure 2:**
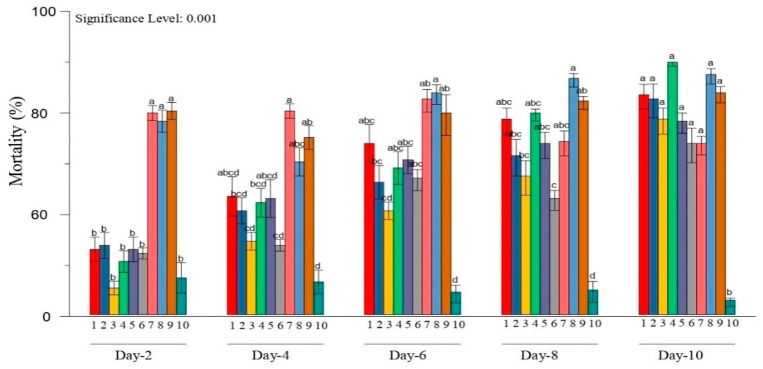
Virulence of entomopathogenic fungi isolates against the *H. puera* larvae, and statistical differences are shown by different letters (Tukey’s test, *p* < 0.001). (1–4) *M. anisopliae* (WnMz1S, NlMz2S, BgMz2S, DhMz4R); (5–7) *M. robertsii* (ArMz3R, ArMz3S, ArMz6W); (8), *M. quizhouense* (ArMz1W); (9), *M. majus* (VjMz1W); (10), Control.

Overall, these results revealed that the three isolates of *M. anisopliae* (DhMz4R), *M. quizhouense* (ArMz1W), and *M. majus* (VjMz1W) showed higher mortality, viz., 94.4 ± 2.03, 92 ± 4.19, and 90.8 ± 3.2 per cent, respectively, against the larvae of *H. puera*. However, there was no significant difference in the pathogenicity of isolates during 10 days after treatment (Figure 2). At 8 days after treatment, there was a significant difference in the pathogenicity among the isolates. The isolate of *M. quizhouense* (ArMz1W) showed significant percent mortality against the 3rd instar larvae of *H. puera* (Figure 2).

### 3.3. Evaluation of Metarhizium sp. Isolates Effected Non-Extracellur Enzymes

The four species of *Metarhizium* sp., viz., *M. robertsii*, *M. quizhouense*, *M. majus* and *M. anisopliae,* were previously screened for pathogenicity [28]. The larvae were evaluated in four concentrations spanning from 1 × 10^3^ to 1 × 10^6^ conidia/mL (Table 2). It was observed that the mortality was increased as the concentration increased. Protease response during the observation (1 × 10^6^ conidia/mL), *M. robertsii* (ArMz3S), *M. quizhouense* (ArMz1W), *M. majus* (VjMz1W), *M.anisopliae* (WnMz1S), and *M. anisopliae* (NlMz2S)showed significantly higher pathogenicity and their percent morality was on par with each other (27.807, 27.063, 27.489, 27.943, and 27.618 percent) (*CD* = 1.050).The lowest percent mortality was shownby the *M. anisopliae* (BgMz2S) isolate.

The percent mortality caused by different isolates of entomopathogenic fungi due to chitinase was significantly different (*CD* = 1.414). *M. quizhouense* (ArMz1W) isolate showed highest percent mortality (28.214%), followed by ArMz3S (27.618%) and ArMz6W (27.489%) at 10^6^ conidia/mL (Table 2).

The results indicate that *M. quizhouense* (ArMz1W) was most effective in causing the mortality against the 3rd instar larvae of *H. puera* through the production of chitinase and protease.

### 3.4. Extracellur Protease Enzymes Activity

**Figure 3:**
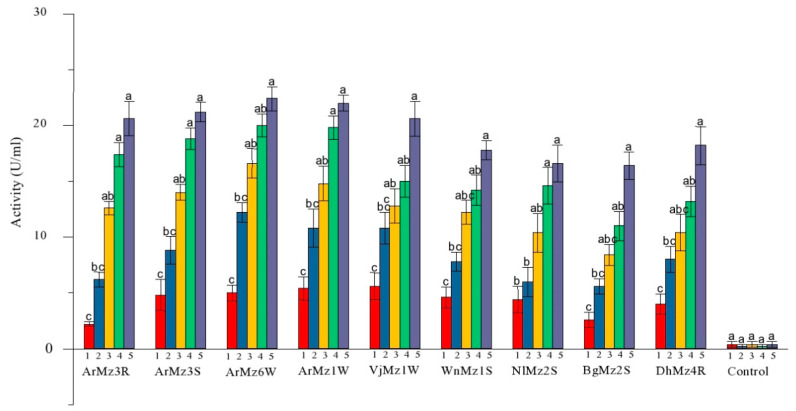
Activities of the proteases (U/mL), Mean±SE in the liquid culture media of the entomopathogenic fungi in the presence of *H. puera* cuticle. Statistical differences are shown by different letters (Tukey’s test, *p* < 0.001). Each color indicating different days of incubation (1st to 5th days).

During the experiment, the effect of protease enzymes on virulence levels was evaluated. Protease activities reached a maximum after 5 days of incubation and decreased thereafter for the control. The protease activity of nine isolates of *Metarhizium* sp. was significantly different from each other (Table 3; Figure 3). Through comparative analysis, the present study demonstrated that the isolates, viz., *M. quizhouense* (ArMz1W) and *M. robertsii* (ArMz6W), showed the highest significant on par protease activity compared with other isolates (22.4 ± 1.0 and 22 ± 0.7 U/mL), respectively, whereas the isolate of *M. anisopliae* (BgMz2S) showed the lowest protease activity.

### 3.5. Extracellur-Chitinases Enzymes Activity

**Figure 4:**
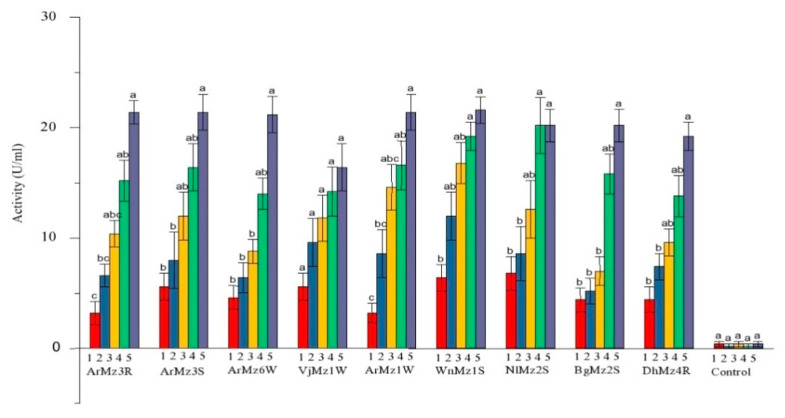
Activities of the chitinases (U/mL), Mean ± SE in the liquid culture media of the entomopathogenic fungi in the presence of *H. puera* cuticle. Statistical differences are shown by different letters (Tukey’s test, *p* < 0.001). Each color indicating different days of incubation (1st to 5th days).

Chitinase activity of *M. anisopliae, M. majus, M. robertsii,* and *M. quizhouense* was varied significantly between the nine pathogenic isolates on 5 days post inoculation in chitin medium (CD(0.01) = 2.684). The comparative analysis demonstrated that the isolates, viz., *M. robertsii* (ArMz3S, ArMz3R, and ArMz1W) and *M. anisopliae* (WnMz1S), showed statistically on par chitinase activity. The chitinase activity was found to be at its maximum during the 5th day of inoculation (Table 4; Figure 4).

### 3.6. Heat Tolerance and Cold Activity

**Figure 5:**
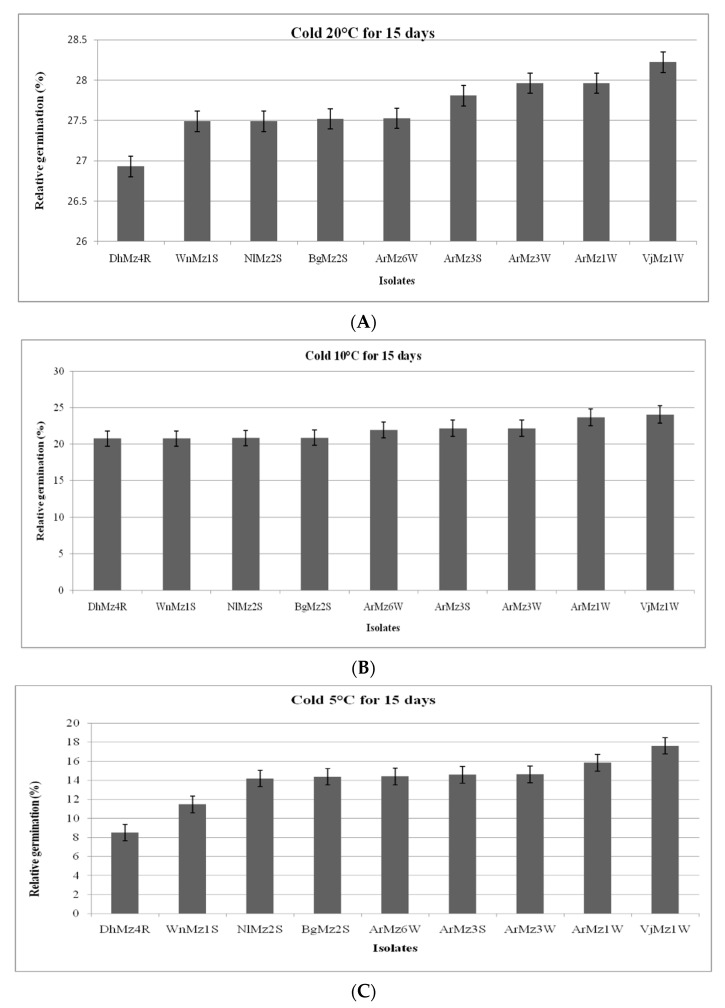
(**A**–**C**): Relative germination (RG) of *Metarhizium* spp. conidia after incubation at 5 °C, 10 °C, and 20 °C for 15 days. (**A**) At 20 °C; (**B**) At 10 °C; (**C**) At 5 °C.

High variability in conidia thermo-tolerance was observed among the *Metarhizium* isolates. However, *Metarhizium* sp. isolates had a maximum germination after being exposed to cold germination temperatures at 20 °C for 15 days in-between (26.92 to 28.22%) for *M. anisopliae* (Dhz14R) and *M. majus* (VjMz1W), respectively. In contrast, the second high variability in cold activity was observed when conidia wasexplored at 10 °C for 15 days, with results ranging from (20.77 to 24.05%) in *M. anisopliae* (DhMz4R) and *M. majus* (VjMz1W). Similarly conidial germination was greatly reduced at 5 °C for 15 days with the exceptions of *M. anisopliae* (DhMz4R) and *M. majus* (VjMz1W), which had approximately lower germination (Figure 5A–C).

**Figure 6:**
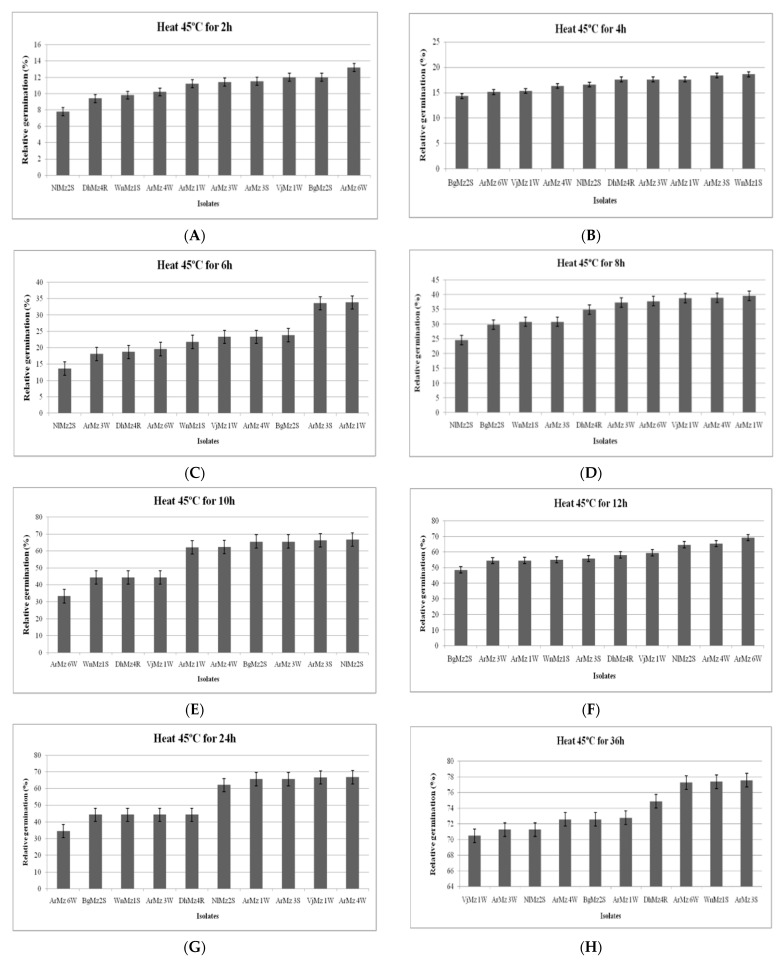

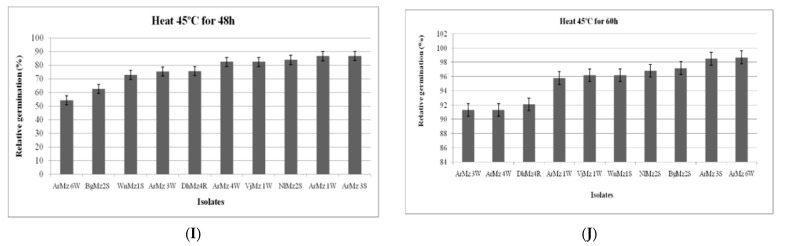
Relative germination of different isolates of *Metarhizium* sp., exposed at 45 °C. Relative germination (RG) of *Metarhizium* spp. conidia exposed for (**A**) 2 h, (**B**) 4 h, (**C**) 6 h, (**D**) 8 h, (**E**) 10 h, (**F**) 12 h, (**G**) 24 h, (**H**) 36 h, (**I**) 48 h, and (**J**) 60 h. RG was calculated in relation to non-heated controls.

**Figure 7:**
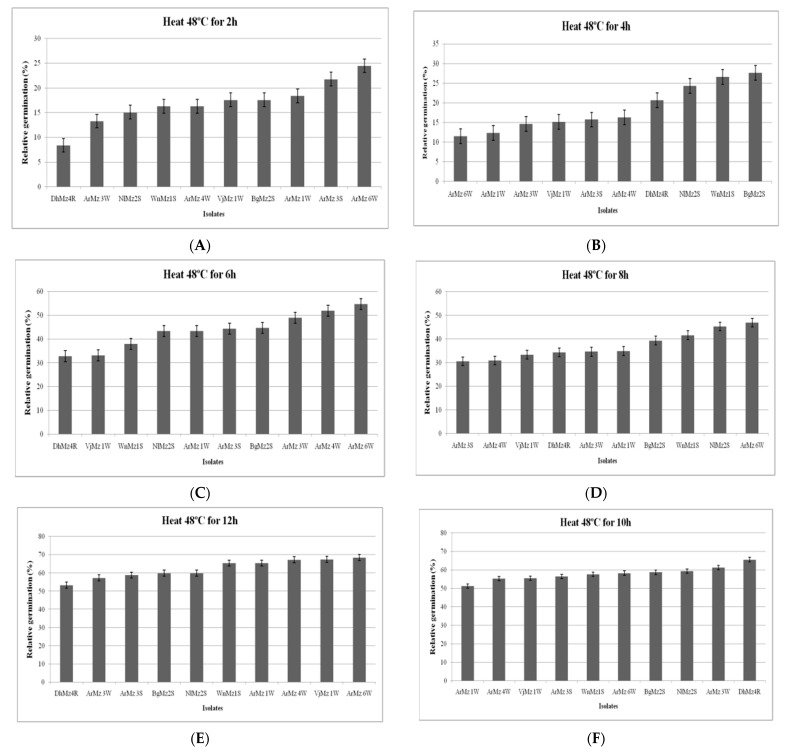

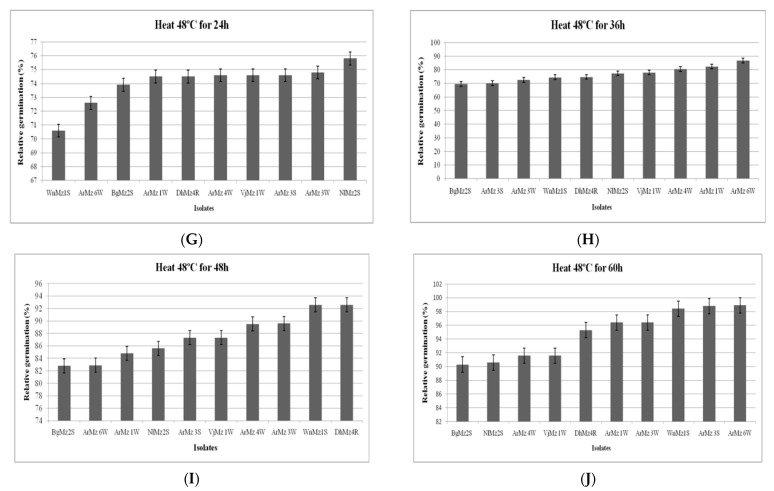
Relative germination of different isolates of *Metarhizium* sp. exposed at 48 °C. Relative germination (RG) of *Metarhizium* spp. conidia exposed for (**A**) 2 h, (**B**) 4 h, (**C**) 6 h, (**D**) 8 h, (**E**) 10 h, (**F**) 12 h, (**G**) 24 h, (**H**) 36 h, (**I**) 48 h, and (**J**) 60 h. RG was calculated in relation to non-heated controls.

Among the 9 *Metarhizium* spp., which were exposed at 45 °C and 48 °C for 2 h, 4 h, 6 h, 8 h, and 10 h, the isolates of *Metarhizium* sp. varied in their conidial germination (Figure 6A–J and Figure 7A–J). All the isolates showed progressive germination in 12 h, 24 h, 36 h, 48 h, and 60 h after exposure at 45 °C. However, at highest exposure (60 h), the isolates, viz., ArMz3S and ArMz6W, showed the highest conidial germination (Figure 6J).It was observed that the conidial germination was high when the incubation time increasedto 48 °C (Figure 7). At the highest exposure (60 h), the isolates, viz., *M. robertsii* (ArMz6W), *M. quizhouense* (ArMz1W), and *M. majus* (VjMz1W), showed the highest conidial germination (Figure 7J).

## 4. Discussion

The identification was done on the basis of morphotaxonomic characters revealed by microscopically inspecting the conidia forming mycelia for conidigenious structure. Each isolate was put into regular host passages using the natural hosts on three species of *M. anisopliae*, *M. robertsii,* and *M. majus* isolate [2,3]. The effect of visible light during mycelia growth factor on the stress tolerance may exhibit an enhanced protein metabolism as suggested by the conidia [7,11,38].

In the present study, nine isolates of *Metarhizium* sp. were characterized for thermal tolerance and cold activity.For cold activity, the isolates were exposed to low temperature, viz., 5, 10, and 20 °C. *M. majus* (VjMz1W) showed higher conidial germination in all the cold temperatures. An earlier study reported that the *Metarhizium* sp., viz., *M. flavoviridae* and *M. frigidium,* were able to tolerate the cold temperature and produced conidia at the lowest temperature of their experiments (5 °C).

The heat tolerance of isolates of *Metarhizium* sp. was tested at the higher temperatures of 45 and 48 °C in different exposure times (2, 4, 6, 8, 10, 12, 24, 36, 48 and 60 h). The temperature tolerance was increased when the exposure time increased. In both of the heat temperatures, *M. robertsii* (ArMz6W) showed high conidial germination at 60 h after exposure. Earlier studies reported that the isolates of *M. anisopliae* var. *acridium* (ARSEF 6421, ARSEF 3614, ARSEF 5736, ARSEF 324, and ARSEF 2341) were most conidial heat tolerant at higher temperatures (45 °C) [6,7,15,40,41].

In the present investigation, chitinase and protease were found to show high correlation with mortality. Earlier literature has been reported that chitinases and proteases were the virulence factors for entomopathogenicity [19,42]. The hydrophobicity of conidia is one of the important parameters influencing fungus-insect interaction as it helps in adhesion of the fungus to the insect cuticle. This plays an important role in providing nutrients before and after the cuticle is penetrated [20,21]. However, chitinase is required only for a brief period during penetration of the host cuticle and is tightly regulated by chitin degradation products [22,23,33]. The fungus produces spores, which, upon germination, produce young germ tubes that penetrate through the cuticle, physically as well as chemically through the enzymes produced, such as proteases, chitinases, and lipases [43,44,45,46,47].

The insecticidal mechanism underlying in the pathogenicity of isolated fungi lies in presence of cuticle degrading enzymes which make paves way for the entry of EPF in the insect body [48,49,50,51,52]. To invade the insect cuticle, which is mainly composed of chitin and several other proteins, entomopathogens must secrete chitinase and protease enzymes [53,54,55,56,57,58]. Mechanism of enzymatic degradation of larval cuticle for hyphal penetrance by this fungus inside larval body is confirmed by the detection of chitinase and protease enzymes thatare secreted by the fungi.

Our study showed that the isolates of *Metarhizium* sp., viz., *M.anisopliae* (DhMz4R), *M. quizhouense* (ArMz1W), and *M. majus* (VjMz1W), showed higher pathogenicity (94.4 ± 2.03, 92 ± 4.19, and 90.8 ± 3.2 percent mortality, respectively) at 10^6^ conidia/mL. Similarly, Ramadevi et al. [59] conducted the bioassay experiment to test virulence of *M. anisopliae* (MIS2 and MIS7) against *H. puera*. They found less percent mortality compared to the present study isolates.

The four species of *Metarhizium* spp., viz., *M. anisopliae*, *M. robertsii*, *M. quizhouense*, and *M. majus,* were obtained from insects infected and soil baiting methods. Therefore, EPF isolates revealed that for different parts of South India, indigenous isolates showed thebest mortality against 3rd instar larvae *H. puera* in the range of 84–92% within 5 days. Our results have indicated that three species, viz., *M. robertsii*, *M. quizhouense*, and *M. majus*, isolates were recorded for the first time and they had comparatively significantly higher mortality rates.

## 5. Conclusions

This study has revealed that *M. anisopliae*, *M. robertsii*, *M. quizhouense,* and *M. majus* are exceptionally effective and can be considered some of the best EPF isolates. Further investigation to control the infestation of teak defoliator *H. puera* should be performed on plantations in South India. The development of a possible new bio-control agent forthe invasive teak defoliator *H. puera* pest is now possible with a better understanding ofthe potential use of indigenous entomopathogenic fungi.

## Figures and Tables

**Table 1:** List of *Metarhizium* spp. Isolates obtained and sequenced for phylogenetic analysis.

Fungi	Type of Forest	Source	Region	Geographical Origin	Accession Number	
					ITS	RPB1
*M.robertsii* ArMz3R	WEF	Insect(*Protaetia aurichalcea*)	Aralam	11′99° N 75′76° E	KU983799	KU680339
*M.robertsii* ArMz3S	WEF	Insect(*P. aurichalcea*)	Aralam	11′99° N 75′76° E	KU983775	KU680335
*M.robertsii* ArMz6W	WEF	Insect (*P. aurichalcea*)	Aralam	11′99° N 75′76° E	KU983797	KU680341
*M.quizhouense* ArMz1W	WEF	Insect(*Eutectona machaeralis*)	Aralam	11′99° N 75′76° E	KU870314	Not yet
*M.majus* VjMz1W	WEF	Insect(*Melolontha guttigera*)	Coorg	12′20° N 75′80° E	KU983771	KU680342
*M.anisopliae* WnMz1S	WEF	soil	Aralam	11′60° N 76′08° E	KU983788	KU680323
*M.anisopliae* NlMz2S	MDF	soil	Bengaluru	12′97° N 77′59° E	KU983785	KU680325
*M.anisopliae* BgMz2S	DDF	soil	Bengaluru	12′80° N 77′57° E	KU983780	KU680320
*M.anisopliae* DhMz4R	MDF	soil	Palakkad	10′77° N 76′65° E	KU983784	KU680328

Wet Evergreen Forest (WEF), Moist Deciduous (MDF), and Dry Deciduous Forest (DDF). ITS—ribosomal internal transcribed spacer; RPB1—DNA-directed RNA polymerase II subunit.

**Table 2:** Virulence of Entomopathogenic fungi isolates against the third-instar larvae of *H. puera*.

Isolates Name	% Mortality of *H. puera* Due Protease				% Mortality of *H. puera* Due Chitinase			
	10^3^ *	10^4^ *	10^5^ *	10^6^ *	10^3^ *	10^4^ *	10^5^ *	10^6^ *
*M. robertsii* ArMz3R	8.499 ^ab^	13.400 ^ab^	24.121 ^a^	26.929 ^b^	9.862 ^b^	12.826 ^e^	19.355 ^de^	23.619 ^c^
*M. robertsii* ArMz3S	11.465 ^a^	14.771 ^a^	24.539 ^a^	27.807 ^a^	8.879 ^b^	19.594 ^a^	20.270 ^cd^	27.618 ^ab^
*M. robertsii* ArMz6W	8.702 ^ab^	13.285 ^ab^	22.481 ^b^	26.371 ^b^	8.350 ^b^	15.728 ^cd^	20.518 ^cd^	27.489 ^ab^
*M. quizhouense* ArMz1W	8.280 ^ab^	14.503 ^a^	24.850 ^a^	27.063 ^a^	13.241 ^a^	19.575 ^a^	23.607 ^ab^	28.214 ^a^
*M. majus* VjMz1W	5.476 ^bc^	13.067 ^ab^	22.160 ^bc^	27.489 ^a^	13.241 ^a^	19.234 ^ab^	25.673 ^a^	27.083 ^ab^
*M. anisopliae* WnMz1S	3.965 ^c^	13.341 ^ab^	21.524 ^cd^	27.943 ^a^	8.813 ^b^	12.109 ^ef^	24.057 ^ab^	26.648 ^b^
*M. anisopliae* NlMz2S	2.573 ^cd^	12.947 ^ab^	20.997 ^d^	27.618 ^a^	8.501 ^b^	14.019 ^de^	17.910 ^ef^	26.916 ^b^
*M. anisopliae* BgMz2S	2.639 ^cd^	12.419 ^abc^	19.333 ^e^	23.333 ^f^	8.879 ^b^	14.111 ^de^	19.965 ^de^	22.704 ^c^
*M. anisopliae* DhMz4R	4.151 ^c^	10.899 ^bc^	19.218 ^e^	25.284 ^e^	9.912 ^b^	17.039 ^bc^	22.265 ^bc^	26.772 ^b^
Control	0.573 ^d^	1.440 ^d^	6.957 ^f^	10.184 ^g^	1.606 ^c^	2.639 ^g^	4.151 ^g^	9.873 ^d^
SE	18.701	7.849	3.186	2.348	27.273	11.393	8.032	3.159
CD(0.01)	4.459	2.478	1.184	1.050	4.400	3.019	2.897	1.414
CD(0.05)	3.072	1.849	0.883	0.783	3.282	2.252	2.161	1.055

Within the same column, mean ± S.E.M. followed by different letters are significantly different from each other followed by adjusted Tukey test. * Spores/mL.

**Table 3:** Activities of protease (U/mL) (Mean ± SE) in the liquid culture media of the entomopathogenic fungi in the presence of *H. puera* cuticle.

Isolates	Proteases Enzyme Activity (U/mL)					Compatative Analysis
	Day-1	Day-2	Day-3	Day-4	Day-5	
ArMz3R	2.2 ± 0.2 ^c^	6.2 ± 0.6 ^bc^	12.6 ± 0.6 ^ab^	17.4 ± 1.1 ^a^	20.6 ± 1.5 ^a^	20.6 ± 3.4 ^b^
ArMz3S	4.8 ± 1.3 ^c^	8.8 ± 1.2 ^bc^	14 ± 0.7 ^ab^	18.8 ± 0.9 ^a^	21.2 ± 0.8 ^a^	21.2 ± 1.7 ^ab^
ArMz6W	5 ± 0.7 ^c^	12.2 ± 0.8 ^bc^	16.6 ± 1.3 ^ab^	20 ± 1 ^ab^	22.4 ± 1.0 ^a^	22.4 ± 3.3 ^a^
ArMz1W	5.4 ± 1.0 ^c^	10.8 ± 1.7 ^bc^	14.8 ± 1.5 ^ab^	19.8 ± 1.0 ^a^	22 ± 0.7 ^a^	22.0 ± 2.4 ^a^
VjMz1W	5.6 ± 1.2 ^c^	10.8 ± 1.4 ^bc^	12.8 ± 1.5 ^abc^	15 ± 1.41 ^ab^	20.6 ± 1.5 ^a^	20.6 ± 3.1 ^b^
WnMz1S	4.6 ± 0.9 ^c^	7.8 ± 0.8 ^bc^	12.2 ± 1.0 ^abc^	14.2 ± 1.3 ^ab^	17.8 ± 0.8 ^a^	17.8 ± 3.1 ^cd^
NlMz2S	4.4 ± 1.2 ^b^	6 ± 1.3 ^b^	10.4 ± 1.7 ^ab^	14.6 ± 1.6 ^a^	16.6 ± 1.6 ^a^	16.6 ± 1.7 ^de^
BgMz2S	2.6 ± 0.6 ^c^	5.6 ± 0.6 ^bc^	8.4 ± 0.92 ^abc^	11 ± 1.30 ^ab^	16.4 ± 1.2 ^a^	16.4 ± 2.2 ^e^
DhMz4R	4 ± 0.8 ^c^	8 ± 1.1 ^bc^	10.4 ± 1.6 ^abc^	13.2 ± 1.3 ^ab^	18.2 ± 1.7 ^a^	18.2 ± 1.4 ^c^
Control	0.4 ± 0.2 ^a^	0.2 ± 0.2 ^a^	0.4 ± 0.24 ^a^	0.2 ± 0.2 ^a^	0.4 ± 0.2 ^a^	0.4 ± 0.9 ^f^
CV						5.714
CD(0.01)						1.745

Within the same row, mean ± S.E.M. followed by different letters are significantly different from each other followed by adjusted Tukey test.

**Table 4:** Activities of chitinase (U/mL) (Mean ± SE) in the liquid culture media of the entomopathogenic fungi in the presence of *H. puera* cuticle.

Isolates	Chitinases Enzyme Activity (U/mL)					Comparative Analysis
	Day-1	Day-2	Day-3	Day-4	Day-5	
ArMz3R	3.2 ± 1.0 ^c^	6.6 ± 1.0 ^bc^	10.4 ± 1.2 ^abc^	15.2 ± 1.8 ^ab^	21.4 ± 1.0 ^a^	21.4 ± 2.4 ^a^
ArMz3S	5.6 ± 1.2 ^b^	8 ± 2.5 ^b^	12 ± 2.1 ^ab^	16.4 ± 2.1 ^ab^	21.4 ± 1.6 ^a^	21.4 ± 3.6 ^a^
ArMz6W	4.6 ± 1.0 ^b^	6.4 ± 1.3 ^b^	8.8 ± 1.0 ^b^	14 ± 1.4 ^ab^	21.2 ± 1.6 ^a^	21.2 ± 3.7 ^ab^
VjMz1W	5.6 ± 1.2 ^a^	9.6 ± 2.1 ^a^	11.8 ± 2.0 ^a^	14.2 ± 2.2 ^a^	16.4 ± 2.1 ^a^	16.4 ± 4.8 ^c^
ArMz1W	3.2 ± 0.8 ^c^	8.6 ± 2.1 ^bc^	14.6 ± 2.0 ^abc^	16.6 ± 2.2 ^ab^	21.4 ± 1.6 ^a^	21.4 ± 3.6 ^a^
WnMz1S	6.4 ± 1.2 ^b^	12 ± 2.1 ^ab^	16.8 ± 1.8 ^ab^	19.2 ± 1.2 ^a^	21.6 ± 1.2 ^a^	21.6 ± 2.7 ^a^
NlMz2S	6.8 ± 1.5 ^b^	8.6 ± 2.4 ^b^	12.6 ± 2.6 ^ab^	20.2 ± 2.5 ^a^	21.2 ± 1.4 ^a^	20.2 ± 3.3 ^ab^
BgMz2S	4.4 ± 1.0 ^b^	5.2 ± 1.1 ^b^	7 ± 1.3 ^b^	15.8 ± 1.8 ^ab^	20.2 ± 1.4 ^a^	20.2 ± 3.3 ^ab^
DhMz4R	4.4 ± 1.1 ^b^	7.4 ± 1.2 ^b^	9.6 ± 1.2 ^ab^	13.8 ± 1.8 ^ab^	19.2 ± 1.2 ^a^	19.2 ± 2.9 ^b^
Control	0.4 ± 0.2 ^a^	0.2 ± 0.2 ^a^	0.4 ± 0.2 ^a^	0.2 ± 0.2 ^a^	0.4 ± 0.2 ^a^	0.4 ± 0.8 ^d^
CV						8.429
CD(0.01)						2.684

Within the same row, mean ± S.E.M. followed by different letters are significantly different from each other followed by adjusted Tukey test.

## Data Availability

All the relevant data are included within the manuscript.

## Abbreviations

EPF	Entomopathogenic Fungi
PDAY	Potato Dextrose Agar with Yeast extract
SDAY	Sabouraud Dextrose Agar with Yeast extract
ITS	ribosomal internal transcribed spacer
RPB1	DNA-directed RNA polymerase II subunit
